# Xenon Blocks Neuronal Injury Associated with Decompression

**DOI:** 10.1038/srep15093

**Published:** 2015-10-15

**Authors:** Jean-Eric Blatteau, Hélène N. David, Nicolas Vallée, Cedric Meckler, Sebastien Demaistre, Kate Lambrechts, Jean-Jacques Risso, Jacques H. Abraini

**Affiliations:** 1 Institut de Recherche Biomédicale des Armées, Équipe Résidente de Recherche Subaquatique Opérationnelle, BP 600 Toulon Cedex 9, France; 2 Centre de recherche Hôtel-Dieu de Lévis, CSSS Alphonse-Desjardins, Lévis, QC, Canada; 3 Université Laval, Département d’Anesthésiologie, Québec, QC, Canada; 4 Normandie-Université, Université de Caen - Basse Normandie, Caen, France; 5 Laboratoire motricité humaine, éducation, sport, santé (LAMHESS), Université de Toulon UFR STAPS, BP 20132, 83957 La Garde, France

## Abstract

Despite state-of-the-art hyperbaric oxygen (HBO) treatment, about 30% of patients suffering neurologic decompression sickness (DCS) exhibit incomplete recovery. Since the mechanisms of neurologic DCS involve ischemic processes which result in excitotoxicity, it is likely that HBO in combination with an anti-excitotoxic treatment would improve the outcome in patients being treated for DCS. Therefore, in the present study, we investigated the effect of the noble gas xenon in an *ex vivo* model of neurologic DCS. Xenon has been shown to provide neuroprotection in multiple models of acute ischemic insults. Fast decompression compared to slow decompression induced an increase in lactate dehydrogenase (LDH), a well-known marker of sub-lethal cell injury. Post-decompression administration of xenon blocked the increase in LDH release induced by fast decompression. These data suggest that xenon could be an efficient additional treatment to HBO for the treatment of neurologic DCS.

Decompression sickness (DCS) is an environmental hazard that can occur when a subject is decompressed from a given ambient pressure to a lower pressure. Neurologic DCS is predominant. In open water divers, the spinal cord is the most affected area, but the brain can be affected too particularly following an unusual rapid decompression^1^. The clinical picture patterns generally vary from minimal sensory abnormalities to severe sensory, motor and/or urinary disorders, but cerebral damage may also occur particularly following an unusual rapid decompression^2^. In contrast, in aviators, the brain is more commonly affected than the spinal cord^3^. Then, the clinical picture mainly includes visual disturbances, vertigo, altered high cognitive function and speech, hemiparesis and unconsciousness^2^,^3^. In both cases, the pathophysiological mechanisms of neurologic DCS are thought to be the consequence from an excessive venous or arterial gas bubble embolization and from the occurrence of stationary tissular gas bubbles originating from the inert gases dissolved in the spinal cord or the brain parenchyma^4^,^5^. Although the consequence of an excessive vascular bubble formation has been quite well documented in animal studies by showing gas bubble-induced vascular ischemia and subsequent ischemia-induced thrombin generation, blood platelet aggregation and coagulation, and immune-inflammatory responses^6^,^7^,^8^,^9^, the effects of stationary gas bubble formation within the tissues, while being of possible critical importance in the production of mechanically-induced excitotoxic processes and subsequent neuronal injury still remains to be demonstrated. Therapeutically, so far, the gold standard treatment of DCS is hyperbaric oxygen (HBO), thought to act primarily by reducing the size of the inert gas bubbles responsible for the occurrence of DCS and by allowing their progressive washout from the body. However, despite state-of-the-art HBO treatment in hyperbaric medical units about 30% of patients suffering neurologic DCS still exhibit incomplete recovery^10^. Taken together these data indicate that further research is needed to improve knowledge and treatment.

Alternatively and interestingly, animal studies in models of excitotoxic and ischemic insults have provided evidence for the neuroprotective effects of the noble gas xenon^11^,^12^,^13^,^14^,^15^. Xenon acts at multiple targets such as the N-methyl-D-aspartate (NMDA) glutamatergic receptor^16^,^17^,^18^, the nicotinic acetylcholine receptor^17^, the TREK-1 potassium channel^19^, and enzymes^20^,^21^. However, neuroprotection by xenon is thought to result from its antagonistic action at the NMDA receptor^14^,^15^, whose activation plays a critical role in excitotoxic neuronal death induced by ischemic insults^22^,^23^. In contrast with prototypical NMDA receptor antagonists, which fail to reach the site of brain injury^24^ and possess their own neurotoxicity^25^,^26^,^27^, xenon crosses the blood-brain barrier and has low blood/gas solubility^28^, conditions that are advantageous in terms of rapid inflow and washout, and reduced risk of adverse reactions^29^,^30^,^31^.

In the present study, given the pathophysiological mechanisms of DCS and the neuroprotective properties of xenon described above, we investigated whether xenon may provide neuroprotection in an *ex vivo* model of neurologic DCS^5^.

## Materials and Methods

Brain slices were drawn from male adult Sprague-Dawley rats (Janvier, Le Genest Saint-Isle, France) weighing 250–280 g, in accordance with the Declaration of Helsinki and the framework of the French legislation for the use of animals in biomedical experimentation as follows: Rats were killed by decapitation, and the brains were carefully removed and placed in ice-cold freshly prepared artificial cerebrospinal fluid (aCSF) containing 120 mM NaCl, 2 mM KCl, 2 mM CaCl2, 26 mM NaHCO3, 1.19 mM MgSO4, 1.18 mM KH2PO4, 11 mM d-glucose, and 30 mM HEPES. Coronal brain slices of 400-μm thickness including the striatum (anteriority: from −1.2 to +2 mm from bregma) were cut using a tissue chopper (Mickie Laboratory Engineering Co., Gomshall, Surrey, UK), and allowed to recover at room temperature for 45 min in freshly prepared aCSF, saturated and continuously bubbled with 100 vol% oxygen.

**Figure 1:**
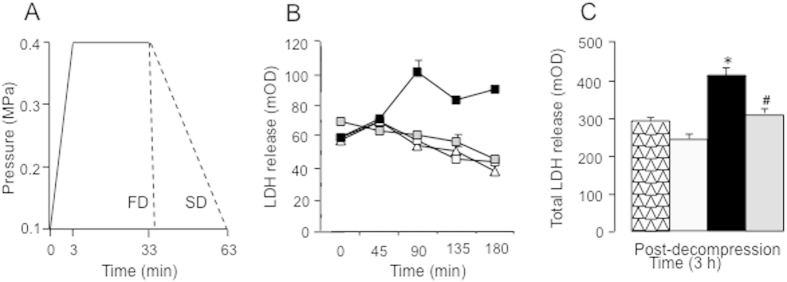
(**A**) Diving profiles of the experiments: Compression was performed at a rate of 0.1 MPa.mim^−1^, and decompression at rate of 0.01 MPa.min^−1^ (slow decompression, SD) or 0.3 MPa.min^−1^ (fast decompression, FD). (**B**) Time course of LDH release and (**C**) total release of LDH over the 3-h post-decompression period. Brain slices exposed to fast decompression + post-decompression medicinal air (◼; *n* = 28) showed a significant increase in LDH release as compared to sham slices maintained at normal atmospheric pressure (△; *n* = 26) and to brain slices exposed to slow decompression and medicinal air (◻; *n* = 28). Administration of post-decompression xenon at 50 vol% suppressed the increase in LDH release induced by fast decompression (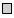; *n* = 14). No significant difference in LDH release was found between brain slices exposed to fast decompression and xenon and both sham slices maintained at normal pressure and brain slices exposed to slow decompression. Error bars are standard error to the mean. If not shown, error bars are included in symbols. Data were compared using the Tukey-Kramer variant of the Tukey’s honestly significant difference method for samples of different size. *P < 0.0001 *vs* sham slices and slices exposed to slow decompression and air; ^#^*P* < 0.0001 *vs* brain slices exposed to fast decompression and air.

After recovery at room temperature, brain slices were incubated in individual vials in a versatile normobaric-hyperbaric chamber, which was placed in an oven at 36 ± 0.5 °C. Temperature was controlled using a temperature probe placed in an empty vial (for technical details about the normobaric-hyperbaric chamber, see ref. 5). Brain slices were first exposed at a pressure of 0.1 MPa (~equivalent to 1 atmosphere absolute) for an additional 45-min period in 1.3 ml of freshly prepared aCSF, saturated and continuously bubbled with medicinal air, composed of 75 vol% nitrogen and 25 vol% oxygen, at a flow rate of 25 ml/min per well. Then, brain slices were incubated in 1.3 ml of freshly prepared aCSF, and compressed with medicinal air to an ambient pressure of 0.4 MPa (~4 atmospheres absolute) at a compression rate of 0.1 MPa.min^−1^; after a steady stay of 30 min at 0.4 MPa, brain slices were decompressed to normal pressure (0.1 MPa) at a decompression rate of 0.01 MPa.min^−1^ (slow decompression rate) or 0.3 MPa.min^−1^ (fast decompression rate). After decompression, brain slices were incubated in 1.3 ml of freshly prepared aCSF, saturated and continuously bubbled at a flow rate of 25 ml/min per well with medicinal air or xenon at 50 vol% (with the remainder being 25 vol% nitrogen and 25 vol% oxygen), shown to provide maximal neuroprotection in *in vitro* and *in vivo* models of ischemic insults^11^,^14^ aCSF was renewed every 45 min, allowing samples to be obtained from the removed solution to assess the time course of lactate dehydrogenase (LDH) released from the brain slices after decompression used as a marker of sublethal cell injury^14^,^32^. Sham slices, instead of being compressed, were incubated in 1.3 ml of freshly prepared aCSF at 0.1 MPa absolute pressure. Compression and decompression profiles and treatments are detailed in Fig. 1A. LDH activity was measured using a spectrophotometer at 340 nm in 50 μL of incubation medium by following the oxidation (decrease in absorbance) of 100 mL of β-NADH (3 mg in 10 ml of PBS) in 20 μL of sodium pyruvate (6.25 mg in 10 mL of PBS) using a microplate reader^32^. LDH effluxes were expressed as a percentage of LDH release in sham slices. The number of slices per condition was n = 14–28. All experimental procedures were approved by the ethics committee of the Institut de Recherche Biomédicale des Armées.

Data are expressed as the mean ± the standard error to the mean, and were analyzed using parametric statistics. Between-group comparisons on total LDH release were performed using parametric ANOVA. Following a significant *F* value, post-hoc analysis was performed using the Tukey-Kramer variant of the Tukey’s honestly significant difference (HSD) method for samples of different size. Level of significance was set up at *P* < 0.05.

## Results

Sham slices maintained to normal atmospheric pressure showed no increase in LDH release over the 3-h duration of the experiment (total LDH release: 275 ± 37 mOD). Likewise, brain slices exposed to high hyperbaric pressure and then to slow decompression also showed no increase in LDH release (total LDH release: 246 ± 53 mOD). This led to no significant difference in LDH release between sham slices and brain slices exposed to slow decompression (HSD values: 42.779 < 47.025, *n.s*.). In contrast, brain slices exposed to high hyperbaric pressure and then to fast decompression – the most important cause of cerebral DCS – showed a sustained increase in LDH release (total LDH release: 415 ± 66 mOD). This resulted in a significant difference in LDH release between brain slices exposed to fast decompression and both sham slices maintained to normal pressure (HSD values: 150.428 > 47.025, *P* < 0.0001) and brain slices exposed to slow decompression (HSD values: 193.207 > 45.725, *P* < 0.0001).

Administration of xenon instead of medicinal air immediately after fast decompression suppressed the facilitating effect of fast decompression on LDH release (total LDH release: 305 ± 14 mOD). This resulted in a significant difference in LDH release between brain slices exposed to fast decompression and xenon and those exposed to fast decompression and medicinal air (HSD values: 147.021 > 55.375, *P* < 0.0001). In contrast, this led to no significant difference in LDH release between brain slices exposed to fast decompression and xenon and both sham slices maintained at normal atmospheric pressure (HSD values: 3.408 < 56.454, *n.s*.) and brain slices exposed to slow decompression (HSD values: 46.186 < 55.375, *n.s*.). All data are illustrated in Fig. 1B,C.

## Discussion

In the present study, we investigated the effect of the noble gas xenon on the release of LDH, known to be a marker of glutamate-mediated sub-lethal cell injury^14^,^32^, using an *ex vivo* model of neurologic DCS^5^.

We found that administration of xenon after decompression blocked the increase in LDH release induced by fast decompression compared to slow decompression. These data are in excellent agreement with previous studies that have clearly demonstrated the neuroprotective action of xenon in *ex vivo* and *in vivo* models of acute hypoxic-ischemic insults^11^,^12^,^13^,^14^,^15^. In brain slices, xenon has been shown to block the increase in LDH release in response to oxygen and glucose deprivation and *in vivo* to dramatically reduce neuronal death that had been induced either by an intrastriatal injection of NMDA or by mechanical and thromboembolic focal brain ischemia^11^,^12^,^13^,^14^,^20^. Because xenon provides neuroprotection through its antagonistic properties at the NMDA glutamatergic receptor^14^,^15^, whose overactivation is well known to play a critical role in neuronal death^22^,^23^, our findings suggest that the NMDA receptor-mediated glutamatergic neurotransmission could play a critical role in the pathophysiological mechanisms of neurologic DCS. Interestingly, this possibility is in line with previous data that have shown a higher DCS incidence in knock-out mice lacking the TREK-1 potassium channel^33^, which is known to maintain an inhibitory input on the NMDA receptor^34^, compared to wild-type mice. Taken together, these results suggest that the use of xenon could allow improving the treatment of neurologic DCS, particularly in the patients who still exhibit incomplete recovery after and despite state-of-the-art HBO treatment in hyperbaric medical units^10^.

So far, the gold standard treatment of DCS is HBO, which is thought to act by reducing the size of the vascular and tissular inert gas bubbles responsible for the occurrence of DCS and thereby to allow their progressive washout from the body. However, recent studies have clearly demonstrated a prothrombolytic action of normobaric and hyperbaric oxygen through activation of endogenous tissue-plasminogen activator^35^,^36^, whose recombinant form is the only treatment of acute ischemic stroke approved to date by the United States Food and Drug Administration and the European Medical Agencies. With little doubt, these prothrombolytic properties of HBO also contribute to the therapeutic efficiency of HBO by breaking up the blood clot induced by vascular gas bubble embolization. Therefore, because xenon is potent inhibitor of tissue-plasminogen activator^20^, it is likely that xenon should be administered after, but not before or during HBO, in order (i) not to inhibit the prothrombolytic action of HBO and (ii) once reperfusion has occurred to provide neuroprotection through its antagonist action at the NMDA glutamatergic receptor.

In conclusion, this study supports that the NMDA receptor-mediated glutamatergic neurotransmission could play a critical role in the pathophysiological mechanisms of neurologic DCS, at least those related to the formation of tissular gas bubbles, and further suggests that administering xenon after HBO therapy could be of potential interest in the treatment of neurological DCS.

## Additional Information

**How to cite this article**: Blatteau, J.-E. *et al*. Xenon Blocks Neuronal Injury Associated with Decompression. *Sci. Rep*. **5**, 15093; doi: 10.1038/srep15093 (2015).
